# Airway Inflammation and Host Responses in the Era of CFTR Modulators

**DOI:** 10.3390/ijms21176379

**Published:** 2020-09-02

**Authors:** Karen Keown, Ryan Brown, Declan F. Doherty, Claire Houston, Michael C. McKelvey, Shannice Creane, Dermot Linden, Daniel F. McAuley, Joseph C. Kidney, Sinéad Weldon, Damian G. Downey, Clifford C. Taggart

**Affiliations:** 1 Airway Innate Immunity Research (AiiR) group, Wellcome-Wolfson Institute for Experimental Medicine, Queen’s University Belfast, Belfast BT9 7BL, Northern Ireland, UK; kkeown02@qub.ac.uk (K.K.); rr.brown@qub.ac.uk (R.B.); d.doherty@qub.ac.uk (D.F.D.); chouston13@qub.ac.uk (C.H.); mmckelvey04@qub.ac.uk (M.C.M.); sfraser04@qub.ac.uk (S.C.); dlinden02@qub.ac.uk (D.L.); s.weldon@qub.ac.uk (S.W.); 2 Wellcome-Wolfson Institute for Experimental Medicine, Queen’s University Belfast, Belfast BT9 7BL, Northern Ireland, UK; d.f.mcauley@qub.ac.uk (D.F.M.); d.downey@qub.ac.uk (D.G.D.); 3 Belfast Health and Social Care Trust, Belfast BT13 1FD, Northern Ireland, UK; joe.kidney@belfasttrust.hscni.net

**Keywords:** cystic fibrosis, inflammation, infection, CFTR modulator

## Abstract

The arrival of cystic fibrosis transmembrane conductance regulator (CFTR) modulators as a new class of treatment for cystic fibrosis (CF) in 2012 represented a pivotal advance in disease management, as these small molecules directly target the upstream underlying protein defect. Further advancements in the development and scope of these genotype-specific therapies have been transformative for an increasing number of people with CF (PWCF). Despite clear improvements in CFTR function and clinical endpoints such as lung function, body mass index (BMI), and frequency of pulmonary exacerbations, current evidence suggests that CFTR modulators do not prevent continued decline in lung function, halt disease progression, or ameliorate pathogenic organisms in those with established lung disease. Furthermore, it remains unknown whether their restorative effects extend to dysfunctional CFTR expressed in phagocytes and other immune cells, which could modulate airway inflammation. In this review, we explore the effects of CFTR modulators on airway inflammation, infection, and their influence on the impaired pulmonary host defences associated with CF lung disease. We also consider the role of inflammation-directed therapies in light of the widespread clinical use of CFTR modulators and identify key areas for future research.

## 1. Introduction

Cystic fibrosis (CF) is a genetic disease caused by mutations in the CF transmembrane conductance regulator (CFTR) gene, resulting in dysfunctional or absent CFTR protein on the apical membrane of epithelial cells. The principal function of the CFTR protein is as a transporter of chloride and bicarbonate ions across epithelial surfaces. In the airway, CFTR also functions as a negative regulator of the epithelial Na+ channel (ENaC) and CFTR dysfunction impacts on pulmonary innate immune responses. Dysfunctional CFTR impairs mucociliary clearance (MCC) and is associated with persistent airway infection and unresolved inflammation, leading to obstructive lung disease and progressive structural damage [1]. This mucoinflammatory cascade is propagated by an intrinsic dysfunction of host immune responses in people with CF (PWCF), leading to a damaging hyperinflammatory state [2,3,4]. As such, the identification of effective anti-inflammatory therapies has been an area of intense research focus.

The arrival of CFTR modulators as a new class of CF therapy in 2012 represented a paradigm shift in how the disease is managed [5], as these small molecules target the upstream underlying defect by improving the expression (correctors), function (potentiators), or quantity (amplifiers) of CFTR protein on the epithelium. There has been ongoing rapid progress in the development of new combination therapies [6,7,8,9,10,11,12]. Following the FDA approval of triple combination therapy (Elexacaftor–Tezacaftor–Ivacaftor) in 2019 [13], around 90% of PWCF have genotypes that are amenable to CFTR modulator therapy, including those with the most common mutation (F508del).

While CFTR modulators are successful in improving epithelial CFTR expression and function, as evidenced by the near-normalisation of sweat chloride levels and improved clinical outcomes, findings to date suggest that they do not prevent the continued decline in lung function, halt disease progression, or decrease the burden of pathogenic organisms in those with established lung disease [14]. Furthermore, it remains unknown whether their restorative effects extend to dysfunctional CFTR expressed in phagocytes and other immune cells. In this review, we examine the pathophysiology of CF inflammation and explore the capacity of CFTR modulators to improve dysregulated inflammation and impaired host responses exhibited by PWCF. We also consider the potential role for anti-inflammatory therapies in light of the widespread clinical use of CFTR modulators and identify some key areas for future research.

## 2. Inflammation in CF

**Figure 1:**
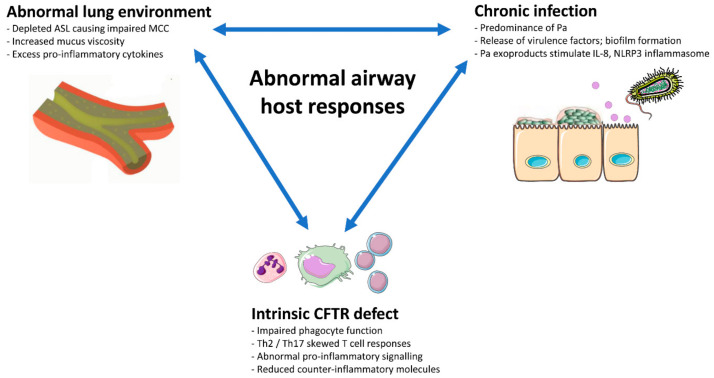
Factors affecting airway host immune responses. Defective cystic fibrosis transmembrane conductance regulator (CFTR) results in dehydration of the airway surface liquid (ASL), which impairs mucociliary clearance (MCC) and host immune responses, thereby permitting a vicious cycle of airway infection (e.g., *Pseudomonas aeruginosa*, Pa), lung damage and a hyperinflammatory state in people with cystic fibrosis (PWCF).

Inflammation is a transient, self-limiting physiological response to injury or infection. However, in CF lung disease, airway inflammation is exaggerated and sustained, playing a central role in disease pathogenesis [2,3,4]. Impaired MCC, airway infection, and abnormal host immune responses all contribute to the dysregulated hyperinflammatory state in PWCF (Figure 1). CF airway inflammation is dominated by neutrophils, which produce reactive oxygen species (ROS), neutrophil extracellular traps, proteases such as neutrophil elastase (NE), and pro-inflammatory mediators, thereby propagating a cycle of progressive tissue damage, immune cell recruitment, and inflammation.

It has been shown that abnormal airway inflammation is present as early as the first few weeks of life [15,16,17] with excess neutrophils and NE present in the airways even in the earliest stages of disease in infants without clinically apparent lung disease [18,19]. This upregulated inflammation results in histopathological abnormalities such as submucosal gland hypertrophy [20] and structural lung disease including mucus plugging and bronchiectatic changes, which can be appreciated radiologically as early as 3 months [17,21]. Inflammation progresses over the first years of life [15,21,22] and persists at abnormal levels throughout the course of the disease [16,23,24]. Elevated baseline inflammation [25] is punctuated with episodic worsening, which may be triggered by a range of factors such as pulmonary exacerbation (PEx), the acquisition of new infective pathogens, CF-related diabetes (CFRD), and poor adherence to therapies.

Although airway infection and impaired MCC certainly worsen inflammation [2,16,17], the causal relationships between infection and inflammation continues to be debated amongst the CF scientific community [26,27]. Studies in newborn CF pigs have shown defective bacterial clearance within hours of birth with an absence of inflammation, indicating that inflammation is generated in response to bacterial stimulation [28]. In contrast, studies in the CF ferret model demonstrate that mucoinflammatory lung disease is present independent of infection, which is sustained despite aggressive bacterial eradication [29]. BAL studies in newborn babies with CF support these findings, showing that inflammation is detected in infants independently of infection [26,27,30], thus favouring that inflammation is a primary consequence of the CFTR defect, rather than purely a response to airway infection

Paradoxically, despite the abundance of neutrophils in the airways, PWCF exhibit impaired host defence, suggesting that CFTR deficiency has negative effects on neutrophil function, indicating either a primary abnormality of the neutrophils themselves or abnormalities in the pro-inflammatory stimuli to which they respond [31,32,33]. CFTR is now known to be expressed not only in epithelial cells but in cells derived from bone marrow, including neutrophils [32] and platelets [34], as well as other cells involved in the innate immune response such as macrophages [35], monocytes [31], lymphocytes [36], and dendritic cells [37]. This intrinsic CFTR defect present in immune cells, together with abnormal signalling mechanisms in PWCF, may contribute to the exuberant inflammatory responses, prolonged inflammation, and delayed clearance of infection [38,39].

### 2.1. Pro-Inflammatory Airway Environment

Epithelial CFTR controls the hydration of the airway surface liquid (ASL) through chloride secretion and the inhibition of ENaC-mediated sodium absorption; this maintains a healthy ASL, enabling an efficient clearance of pathogens. However, in CF, impaired chloride secretion and excess sodium absorption cause dehydration of the mucosal surface, leading to mucus accumulation, plugging of the small airways, and airway obstruction. Loss of the ASL barrier leaves mucosal surfaces exposed and more susceptible to the damaging effects of chronic infection and toxic inflammatory products. Acidification of the ASL through reduced CFTR-mediated bicarbonate transport [33] impairs phagocyte function [40] and ciliary beat frequency [41], adding to the burden of uncleared pathogens and mucoinflammatory products within the airways. Furthermore, mucus viscosity is altered by the abundance of free neutrophilic actin and long-stranded DNA [42], as well as increased IL-1α and IL-1β mucin stimulation (MUC5B and MUC5AC) [43], making it more difficult to clear from the airways. The cumulative result of these factors is an intense mucoinflammatory airway environment that causes progressive mucus obstruction and progressive bronchiectasis [44].

The extracellular airway environment is rich in proteases, which are released by inflammatory cells including neutrophils, macrophages, and epithelial cells [45]. These proteolytic enzymes directly contribute to the pathophysiology of lung disease through degradation of the elastin and collagen, leading to structural damage and bronchiectasis. In addition to direct structural damage, proteases contribute to the mucoinflammatory environment through several other functions: the increase of pro-inflammatory signalling, stimulation of mucin production, activation of ENaC channels, degradation of cognate anti-proteases, and cleavage of residual CFTR protein [46,47,48,49,50,51,52]. Given the neutrophilic nature of CF airway inflammation, neutrophil serine proteases, in particular NE, which correlates directly with decline in FEV_1_ (forced expiratory volume in one second) [4,53,54] and predicts the development of bronchiectasis [22], has received the most attention as a potential target for anti-inflammatory therapies [45,55]. However, as NE inhibition has not yet proved to be effective in clinical studies, the inhibition of other emerging proteases, or combinations of proteases, will need to be investigated as potential targets such as cathepsin S, cathepsin C, and matrix metallopeptidase-12 (MMP-12) [45,56,57,58,59].

Recurring infection upregulates airway inflammation, drives PEx, and is a central factor in the progression of CF lung disease [60,61,62]. The early CF airway is primarily colonised by *Staphylococcus aureus* and *Haemophilus influenzae*, which gives way to reduced species diversity and predominance of the Gram-negative organisms Pa and *Burkholderia* spp. [63,64]. The abnormal airway environment increases host susceptibility to chronic airway infection with these and other pathogens, which is facilitated by abnormal chlorination and impaired microbial killing by CFTR-deficient neutrophils [32]. In addition, CF pathogens display a range of phenotypic and genetic adaptations over the course of chronic infection, which enable their persistence within the CF lung. These include the release of toxic exoproteins, mutation into resistant strains, and the development of biofilms that create a physical barrier to the host immune clearance. The CF inflammatory response to infection is more intense that in non-CF subjects [65], in particular to Pa, which activates the NLRP3 inflammasome through the release of lipopolysaccharide (LPS) [66] and other pro-inflammatory exoproducts that stimulate NF-κB and IL-8 production [67,68].

### 2.2. Abnormal Immune Cell Responses

Absent or mutated CFTR impairs several aspects of neutrophil function, including faulty targeting of CFTR to the appropriate subcellular site, poor chlorination of phagocytosed bacteria, reduced sphingosine-dependent microbicidal ability, delayed apoptosis, and over-exuberant pro-inflammatory responses to acute insults such as LPS [32,69,70,71,72,73,74]. Greater numbers of neutrophils were present in the bronchoalveolar lavage (BAL) fluid of CF mice following Pa challenge as compared to wild-type (WT) mice, which correlated with neutrophilia and elevated IL-17 in BAL fluid and sputum of PWCF during PEx [75]. Degranulation is also impaired, with CF neutrophils releasing more primary granule contents, such as NE [76], and fewer secondary and tertiary granule contents, such as the immunomodulating, antimicrobial glycoprotein lactoferrin [69], which may account for some of the lung tissue remodeling and altered antimicrobial abilities displayed by CF neutrophils.

Macrophages, the resident airway phagocyte, are usually among the first cells to encounter inhaled pathogens and have also been shown to exhibit abnormal function in CF. Defective phagocytosis has been demonstrated in CFTR^−/−^ mice [77,78], where alveolar macrophages have been shown to phagocytose and generate an oxidative burst, but they cannot effectively kill the bacteria [78]. This study demonstrated that lysosomes from CFTR-null macrophages failed to acidify to a pH less than 5, likely as a result of alterations in Cl^−^/HCO_3_^−^ concentrations, compromising bactericidal activity. In addition, defective polarisation to anti-inflammatory M2 macrophages has been reported, with a number of factors suggested to contribute to the M1/M2 polarisation status of macrophages, including the expression levels of IL-4 and IL-13 receptors, IL-4Rα, and IL-13Rα1 [79].

PWCF also exhibit disruption in T- and B-lymphocyte cell responses [36,80,81,82,83,84]. T cell populations are skewed toward inflammatory Th2- and Th17-dominated phenotypes due to intrinsic predisposition and in response to environmental factors such as inflammatory signals and cytokines [80,81,82,84]. Regulatory T cell numbers are significantly reduced in PWCF [84], and they demonstrate a reduced ability to suppress conventional T cell proliferation, which is further impaired during Pa infection [84]. It has also been shown that human and murine CF naive T cells differentiate to the Th17 phenotype more quickly in response to Th17 stimulants [85]. In B cells, a lack of functional CFTR is associated with a reduced ability to produce λ light chain upon stimulation, potentially impairing the B cell response against Pa [36].

Dendritic cells (DCs) play an important role in the initiation and regulation of immune responses. As compared to DCs from WT mice, CFTR^−/−^ mice show delayed differentiation and a reduced expression of caveolin-1, which may contribute to inflammation [86] and the disruption of factors involved in membrane structure and lipid metabolism [37]. These and other unidentified intrinsic abnormalities may contribute to the immune response characteristic of CF, and they are likely to be exacerbated by pro-inflammatory priming that comes from the pathogen as well as the pathogen-associated molecular patterns (PAMP)/damage-associated molecular patterns (DAMP) and cytokine-rich environment of the CF lung.

Platelets have also been shown to function abnormally in PWCF and are gaining recognition for their role in CF airway hyperinflammation [87]. Firstly, compared to healthy controls, platelets from PWCF produce 40% less of the anti-inflammatory lipoxin A_4_ [34]. Secondly, transient receptor potential channel (TRPC) 6-dependent platelet activation, due to abnormal TRPC6-dependent Ca^2+^ influx, leads to aberrant platelet activation [88] and dysregulated platelet responses. This has been demonstrated in F508del mutated mice in whom the instillation of intratracheal LPS produced a severe thrombocytopenia compared to WT mice. Interestingly, the blockade of platelet activation with aspirin significantly reduced the inflammation [89].

The recent characterisation of immune activation profiles using a single-cell transcriptome analysis of immune cells in CF sputum demonstrated a shift from alveolar macrophage dominant to activated neutrophils and macrophages and an immature pro-inflammatory archetype [90].

### 2.3. Impaired Signalling Mechansims

An intrinsic hyperinflammatory phenotype characterised by elevated IL-8 secretion, increased NF-κB, p38/ERK MAPK activation and pro-inflammatory cytokine expression in response to stimuli has been demonstrated in CF airway epithelial cells in vitro [91,92,93]. This was elegantly demonstrated in an experiment using human CF tracheal grafts implanted into animal models, which were shown to release excessive IL-8 [81] as compared to non-CF tracheal grafts [94]. Furthermore, epithelial Nrf2 antioxidant responses were reduced, leading to elevated intracellular H_2_O_2_ and enhanced pro-inflammatory signalling [95].

A pro-inflammatory signature is also observed in CF macrophages, which have higher TLR4 expression and elevated levels of pro-inflammatory mediators such as TNF-α, IL-1β, and IL-8 [96,97,98,99,100]. In addition, endoplasmic reticulum (ER) stress was detected in monocytes and M1 macrophages in PWCF, and it was associated with an increased metabolic state and an exaggerated production of TNF-α and IL-6 [101]. Altered neutrophil immunometabolism and the subsequent activation of the NLRP3 inflammasome induced by bacterial LPS that produced increased levels of IL-1β, which correlated with neutrophil burden and disease progression [66,102,103].

Recent work by Zhang and colleagues applied transcriptomics to analyse gene expression and microRNA (miRNA)–mRNA networks to distinguish immune cell subsets and identify key inflammatory signalling pathways in PWCF [104]. The analysis of peripheral blood cells showed the downregulation of genes involved in immune cell function, pathways involved in the cellular recognition of bacteria and viruses, and an abundance of miRNAs that target cytokine production genes including IL-8, IL-1β, and TNF-α [104].

### 2.4. Impaired Counter-Inflammatory Mechanisms

Along with the increased influx of neutrophils and other immune cells, impaired function of these cells, and aberrant pro-inflammatory pathways, the lungs of PWCF also exhibit an inability to resolve inflammation appropriately due to deficient or downregulated counter-inflammatory mechanisms. These include the reduced secretion of the anti-inflammatory cytokine IL-10 from CF macrophages [96,105], reduction of the immunomodulator nitric oxide (NO) [106,107], suppressed levels of the anti-neutrophilic molecule lipoxin A4 [108], and MMP-9 cleavage of surfactant protein D [109,110]. Additionally, the endogenous lipid mediator resolvin RvD1, which is a potent regulator or resolution, has reduced receptor expression in CF macrophages and epithelial cells, leading to sustained lung inflammation and uncleared infection [111,112].

Furthermore, intense airway neutrophilia overwhelms the cognate antiprotease capacity, leading to a protease/anti-protease imbalance, which directly contributes to the progression of lung disease [113,114,115].

## 3. Effects of CFTR Modulators on Inflammation

The effects of CFTR modulators on inflammation in general, and the impact they have on intrinsic defects of the innate immune response in PWCF, are thus far under-characterised. The effects of CFTR modulators in clinical trials have focused on pulmonary function, PEx, and quality of life measures, which are important clinical outcomes [6,7,8,9,10,11] and are also required for regulatory approval [116]. Furthermore, existing published research is mainly limited to the effects of the potentiator, ivacaftor (IVA), with little data relating to the other modulator therapies available as yet.

Hisert et al. demonstrated that sputum inflammatory markers decreased significantly in the first week of IVA treatment in patients with G551D mutations, with a continued decline in IL-8, IL-1β, and NE over the subsequent two years [117]. These improvements were accompanied by clinical improvements in FEV_1_ and mucus plugging on high-resolution CT (HRCT). In contrast, a multicentre prospective cohort study of 14 adults and children treated with IVA in the GOAL study (G551D Observational Study) showed no significant change in sputum inflammatory markers, despite significant improvements in FEV_1_ and reduction in sweat chloride [118].

However, a recent study by Jarosz-Griffiths et al. examined the inflammatory effects of CFTR modulators in monocytes isolated from clinically stable patients homozygous for Phe508del CF mutation [119]. Using LPS/ATP stimulation to activate NLRP3, they reported a reduction in IL-18 following the in vitro application of ivacaftor/lumacaftor (IVA/LUM) and ivacaftor/tezacaftor (IVA/TEZ), but a reduction in IL-1β levels was found only with IVA/TEZ. These findings were confirmed in patients following commencement of treatment in the clinic, where again, only IVA/TEZ reduced IL-1β levels in serum. Similarly, after three months of patient treatment, LPS-ATP stimulated the secretion of IL-1β remained unchanged in cells exposed in vivo to IVA/LUM, but it was significantly reduced in cells from patients receiving IVA/TEZ. Furthermore, IL-10 levels were increased in both studies, suggesting that CFTR modulator combinations exhibit anti-inflammatory effects [119].

Although these data could indicate that CFTR modulators improve airway inflammation, results are inconsistent, and studies to date have included only small numbers of participants. Furthermore, it is unclear whether observed effects are due to directly improved host responses or through general improvement of the airway environment. Indeed, patients with the G551D mutation showed marked improvements in whole lung, central, and peripheral MCC at 1 and 3 months after starting treatment with IVA, which correlated with lung function [117]. Other studies have shown reductions in lung clearance index (LCI) with CFTR modulation [120], indicating reduced mucus plugging and improved air trapping [121,122]. A large multicentre study assessing inflammatory and clinical outcomes in Kaftrio (known as Trikafta in the United States; ivacaftor/tezacaftor/elexacaftor) treated patients is underway, which should give a clearer picture of the modulator effects on inflammation [123].

### 3.1. Effects on Immune Cells

**Figure 2:**
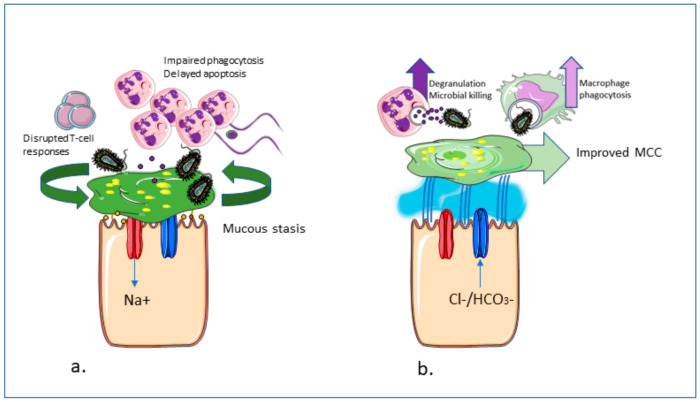
Potential effects of CFTR modulators on inflammation and host responses in cystic fibrosis (CF). (**a**) In CF airways, depleted airway surface liquid (ASL) and poor mucociliary clearance (MCC) leads to mucus adherence to exposed epithelial surfaces. As a result, PWCF have increased susceptibility to, and reduced clearance of, pathogens as well as dysregulated host responses such as impaired neutrophil and macrophage phagocytosis and delayed neutrophil apoptosis. (**b**) With CFTR modulator therapy, restoration of the ASL barrier may be associated with improved MCC and epithelial integrity, increased microbial killing, and clearance due to the restoration of host defence mechanisms, including leukocyte phagocytic function.

While modulators have a positive influence on MCC and are likely to attenuate airway inflammation by this mechanism, CFTR modulators may be able to improve the intrinsic CFTR related immune abnormalities (Figure 2). Several ex vivo studies have evaluated individual cell responses to CFTR modulators.

#### 3.1.1. Neutrophils

One of the earliest ex vivo studies by Pohl et al. demonstrated that treatment with IVA could normalise cytosolic ion levels in neutrophils, restore the capacity to degranulate, and improve microbial killing [69]. Other studies have revealed that either directly or indirectly, CFTR modulators could successfully alter the activation state of neutrophils and reduce cholesterol-mediated neutrophil adhesion [109,124]. Importantly, Gray et al. showed an increased apoptosis of neutrophils from patients treated with IVA, suggesting that modulators may also reverse the damaging pro-survival neutrophil phenotype exhibited in CF, although a direct association between CFTR function and apoptosis pathways has not yet been elucidated [125]. Timely apoptosis is an important anti-inflammatory process that limits the toxic potential of the large number of leukocytes present in the CF lung. Considering what is known about the role of CFTR in the function of neutrophils and the impact of early CFTR modulator studies, it is likely that CFTR modulators do have direct effects on neutrophils, thereby tempering inflammation, but further work is needed to elucidate these effects.

#### 3.1.2. Macrophages

Although CFTR expression is lower in macrophages compared to that observed in some other cells, its expression and function appears to respond to CFTR modulators. Macrophages from PWCF on IVA therapy demonstrate improved phagocytosis and killing of Pa to near-normal levels [126], as well as decreased apoptosis, indicating improved macrophage stability in response to CFTR modulation [126]. However, the combination of LUM/IVA diminished the phagocytic response of both CF and non-CF subjects. Similarly, in another study, Barnaby et al. showed that LUM restored defective monocyte-derived macrophages (MDM) phagocytosis and the killing of Pa to levels comparable to wild-type CFTR MDM [127]. However, the addition of IVA negated this effect and led to the reduced ability of LUM to stimulate phagocytosis, while both IVA and LUM/IVA reduced the secretion of pro-inflammatory cytokines, including IL-6, IL-8, TNF-α, IFN-γ, and GM-CSF. These studies indicate the possible beneficial effects of single agents on phagocytosis, which may be undermined by combination treatments. Further research is needed to better understand differential effects of single and combination modulator compounds.

It has also been shown that CFTR modulators influence the inflammatory responsiveness of macrophages. Proteome analysis of monocyte plasma proteins revealed IVA-associated reductions in inflammatory proteins (S100A9, MX1, and HLA-B) and an increase in proteins involved in monocyte migration (ENO1 and PFN1), all of which could be associated with a dampened IFN-γ response [128]. Follow-on studies revealed that IVA could reduce the responsiveness to IFN-γ in peripheral blood CF monocytes [129]. As IFN-γ has a role in immune modulation and antimicrobial activity, IVA may regulate bacterial burden and inflammation through decreased IFN-γ signalling in CF monocytes. Treatment with IVA reduced the secretion of IL-6, TNF-α, and IL-12 levels to that seen in non-CF cells, whereas LUM reduced IL-6 only in CF MDM [126]. Furthermore, Bratcher et al. showed a reduction in circulating inflammatory makers and dampened stimulation-induced changes in CD13b and CXCR2 after IVA treatment [130].

#### 3.1.3. Lymphocytes and the Adaptive Immune Response

A recent study documented the effects of LUM/IVA therapy over a 12-month period in a small cohort of PWCF homozygous for F508del [131]. After one month of treatment, the number of total white blood cells did not significantly decrease [131]; however, the composition of lymphocyte subsets was not considered. Therefore, alterations in the pattern of the adaptive immune response were not determined. To our knowledge, the only study considering the effect of CFTR modulators on lymphocyte subsets has been reported as an abstract by Kopp and colleagues [132]. The investigators compared peripheral blood lymphocyte subsets of PWCF receiving the CFTR modulators IVA, LUM/IVA, TEZ/IVA, or no treatments with healthy controls [132]. Compared to PWCF on other CFTR modulators, those with IVA-responsive genotypes exhibited a resolution of T helper cell, cytotoxic T cell, and B-lymphocyte deficits, but there was little change in the number of regulatory T cells [132]. The data from this study has yet to be published, but the preliminary finding of restoration of adaptive immune cell deficits highlights the insights that can be gleaned from differentiating between lymphocyte subsets.

RNA-seq analysis of whole blood from PWCF compared to non-CF individuals revealed alterations in the expression of gene transcripts involved in innate and adaptive immunity pathways [133]. Six-month administration of LUM/IVA resulted in a decreased expression of cell death-related genes post-treatment, but an over-expression of inflammation-related transcripts as well as suppression of T cell and NK cell transcripts persisted [133]. LUM/IVA may exert minor effects on immune responses in older PWCF with established lung disease; however, the effects in younger patients with less established disease are not yet known [133].

The effect of CFTR modulators on cells of the adaptive immune system has received little attention in the literature to date. Studies that do acknowledge the adaptive arm of the immune response do not typically differentiate between lymphocyte subsets, such as pro- and anti-inflammatory T cell subsets. Further study is required to elucidate the effect of CFTR modulators on lymphocyte subsets and activities, such as the ability to produce an effective antibody response upon vaccination.

#### 3.1.4. Airway Epithelial Cells

The effect of CFTR modulation on epithelial immune responses have also been demonstrated. Firstly, Nrf2 activation in human bronchial epithelial (HBE) cells from PWCF was increased by the correctors LUM and TEZ, which is an effect found to be mediated by CFTR modulator-induced increases in CREB-binding protein (CBP), which can interact with and activate Nrf2 [134]. Considering the role of Nrf2 in inflammatory signalling, the correction of Nrf2 regulation has the potential to modulate the aberrant inflammatory signalling in the CF airways. In a further study of LUM/IVA, accelerated epithelial repair rates and enhanced transepithelial electrical resistance (TEER) were observed in the presence of Pa exoproducts [135], indicating the potential for enhanced reparative and regenerative processes in the airways as a result of the restoration of epithelial CFTR function.

Ruffin et al. demonstrated that the treatment of CF HBEs with LUM/IVA prevented p38 MAPK phosphorylation and CXCL1/2 mRNA expression induced by exoproducts from a Pa clinical isolate, whilst also reducing CXCL8 mRNA expression to levels comparable to uninfected cells [136]. However, a prior study reported that this drug combination failed to reduce constitutive or Pa laboratory strain PAO1-stimulated IL-6 and IL-8 secretion by CF HBEs [137]. It is important to note that key methodological differences may have contributed to the discordant results in these studies, including different cell sources and culture protocols, drug concentrations, and infection protocols.

These studies show that as well as improving their primary function as airway fluid regulators, CFTR modulators also have effects on epithelial immune function with evidence that they directly downregulate inflammatory responses. The effects of CFTR modulators on airway epithelial immune function, as well as their influence on redox balance, lipid metabolism, pathogen interactions, and epithelial repair [138,139,140,141,142,143] warrant further evaluation given the clear effects of modulator therapy on epithelial cell fluid regulation.

#### 3.1.5. Platelets

It has recently been shown that a loss of CFTR dysfunction function in platelets produces exaggerated inflammatory responses after intratracheal challenges through TRPC 6-dependent platelet activation, which have been shown to improve in subjects receiving modulator therapy (LUM/IVA) with a partial restoration of CFTR function in platelets [144]. These findings highlight that much is yet unknown about the wider effects of CFTR modulator therapies on local and systemic host responses.

### 3.2. Other Effects

Abnormalities in fatty acid metabolism in PWCF can cause an increased production of the pro-inflammatory eicosanoid arachidonic acid, which has numerous downstream products including prostaglandins and leukotrienes and its by-product, urinary prostaglandin-E metabolite [145]. IVA therapy in individuals with at least one gating mutation resulted in significantly decreased urine prostaglandin-E metabolite without detectable changes to the overall fatty acid profile [146], suggesting a unique mechanism by which IVA may reduce inflammation, independent of its effects in the lungs.

Beyond the effects on cellular responses, much is yet unknown about the effects of CFTR modulators on dysregulated signalling pathways and disrupted homeostatic mechanisms such as proteostasis and autophagy, which are processes responsible for the clearance of impaired proteins, lipids, and organelles. Uncleared, these substrates aggregate in the mucosal tissues and lead to the accumulation of toxic protein products, ROS production, and increased inflammatory–oxidative stress [147]. CFTR contributes to the orchestration of these mechanisms; thus, the restoration of CFTR function (via CFTR modulators) may improve these vital processes, but this have yet to be explored. Furthermore, the effects of CFTR modulators on dysregulated intracellular communication mechanisms, including miRNAs that negatively regulate CFTR and contribute to abnormal immune responses, are unknown [148].

Overall, research to date suggests that CFTR modulation can alter CFTR expression, affect cell stability, improve phagocytosis and killing of bacteria, and reduce pro-inflammatory responses of key cellular and signalling mechanisms involved in abnormal innate immune function associated with CF. However, uncertainty remains as to whether these abnormalities are a primary consequence of CFTR dysfunction or if they have been induced secondarily by the altered disease environment. It is still unclear whether in vitro findings accurately represent what actually occurs in PWCF treated with CFTR modulators.

## 4. CFTR Modulators, the Microbiome, and Airway Infection

Reduced bacterial diversity in PWCF is typically associated with increasing age; however, young children with CF have decreased airway microbial diversity compared to healthy children of the same age [148]. In children with CF, modulators have been shown to improve bacterial diversity to a state similar to that of healthy individuals (IVA and TEZ/IVA) [149], with similar findings in adults with the G551D mutation taking IVA [117,118,150]. However, only small numbers of patients have been studied, and as established microbial communities may take time to adjust, larger longitudinal microbiome studies are needed to assess the long-term effects on the microbiome.

Hisert et al. have shown that Pa burden in chronically infected patients fell within 48 h after initiation of IVA, with a 10-fold reduction in density after 7 days [117]. However, none of the 8 chronically infected patients studied became consistently culture negative, and the Pa density increased again in the second year of treatment. In the GOAL study, Rowe et al. followed up 153 patients with G551D mutation 6 months after starting treatment with IVA. Fewer patients isolated Pa from respiratory specimens compared to pre-treatment stage; however, the majority of subjects were not chronically infected with Pa [118]. A more detailed subgroup microbial analysis revealed that although the relative abundance of CF pathogens trended downwards, neither total bacterial load nor Pa load changed significantly following IVA treatment [118], although this analysis was limited to only 14 patients. The reason for these inconsistent responses to IVA is not clear but it may suggest variable improvements in MCC rather than direct antimicrobial effects of IVA.

However, in vitro studies indicate that IVA exhibits direct antimicrobial effects towards certain pathogens, in particular *S. aureus* and *Streptococcus pneumonia*, while activity against Pa is limited [151]. Consistent with these findings, Payne et al. showed bactericidal activity of IVA against CF clinical isolates of *Streptococcus* spp. and bacteriostatic activity against *S. aureus* but no effect on Pa [136]. It has been proposed that the antimicrobial effects of IVA toward certain pathogens may be attributed to the presence of a quinolone ring in its structure [137]; however, the inhibition of DNA gyrase and topoisomerase IV, the mechanism of action of quinolone antibiotics, was not observed with IVA [138]. Furthermore, as quinolones have significant Gram-negative antimicrobial activity, a clear anti-pseudomonal effect would be expected.

A synergistic effect between CFTR modulators and several antimicrobial agents commonly used in CF, including ceftazidime, tobramycin, ceftriaxone, vancomycin, trimethoprim–sulfamethoxazole, moxifloxacin, linezolid, and polymyxin B has been observed [136,138,139,151]. While the mechanisms behind this are largely unexplored, it is possible that permeabilisation of the Gram-negative outer membrane by antibiotics such as polymyxin B allows modulators to enter the cell and exert their antimicrobial activity.

CFTR modulator-mediated improvements in MCC and phagocytic activity are likely to account for some of the improvements in bacterial burden resulting in a shift toward a healthier airway microbial composition. However, direct antimicrobial action against certain pathogens cannot be excluded. Antifungal activity of IVA has also been shown in vivo with reduction in *Aspergillus* colonisation [152], and it has recently be shown that CFTR modulators dampen *Aspergillus*-induced ROS production [153], indicating a potential role to improve *Aspergillus*-induced inflammation in CF.

Furthermore, as CFTR modulators have been shown to modify the gut milieu to favour a healthier microbiota [140], they may have broader constitutional effects on host–microbe interactions, which could lead to improvements in extra-pulmonary CF morbidity. While these studies include only small sample sizes, they indicate that CFTR modulators influence both the composition of airway microbiota and exert some degree of antimicrobial activity against certain CF pathogens. Further studies to understand these, as yet, unidentified direct and indirect mechanisms of CFTR modulator effect are needed, as they may significantly influence disease progression and the post-modulator disease phenotype.

## 5. Conclusions and Future Directions

As CFTR modulator therapies are reaching more PWCF, their clinical effects are becoming clearer. However, our understanding of the full extent of their therapeutic effects are limited by the lack of data relating to inflammatory and immunological outcomes. Given that IVA was the first CFTR modulator to receive regulatory approval, it is not surprising that most studies have focussed on its effects in patients with gating mutations. However, the effects demonstrated by a single agent in gating mutations alone may not be generalisable across different modulator formulations and genotypes, as individual responses are subject to a range of factors including pharmacokinetics, residual CFTR function, and disease severity at initiation. To date, little is known about the capabilities of CFTR modulator therapies to restore CFTR function in non-epithelial cells. Furthermore, CF is a multisystem disease, and as we have highlighted, CFTR is expressed widely in cells of the innate immune system and many other cell types. There is evidence that modulators improve pancreatic exocrine and endocrine function, CF liver disease, bone disease [141], and have effects on haemopoietic cells [142,144], indicating that they have a broad scope of action across a range of cell types, supporting their potential for direct effects on defective CFTR expressed in immune cells.

The existing data that we have summarised in this review shed light on the multiple mechanisms by which CFTR modulators may attenuate the hyperinflammatory CF airway environment. Ex vivo studies have revealed direct effects of CFTR modulators on the function of neutrophils [69,109,124,125], macrophages [126,127,130], lymphocytes [131,132,133], epithelial cells [134,135], and their signalling pathways [124,128,132]. There have been additional effects of CFTR modulators demonstrating improvements in MCC including LCI, mucus plugging, and air trapping [117,121,122], as well as effects on ciliary function [143]. However, despite these findings, uncertainty remains as to whether these effects are directly attributable to modulator effects on target cells or whether they are secondary to modification of the diseased airway environment. Separation of the direct and indirect effects CFTR modulators will be vital in understanding their mechanisms of action, which will inform development of newer agents and will be vital in characterising the inflammatory phenotype of post-modulator CF lung disease.

Despite evidence of feasible direct immunomodulatory mechanisms, studies thus far show only modest or no improvements in markers of airway inflammation and a non-sustained effect on microbial burden in patients receiving CFTR modulator treatment. However, a major limitation of existing clinical studies is the lack of stratification of patients based on age and disease severity at time of initiation. Timing is likely to be a key determinant of modulator effects on inflammation, as those with established bronchiectasis and embedded airway infection are likely to continue to exhibit airway inflammation. Furthermore, although CFTR modulators may improve the regenerative properties of epithelial cells and macrophages [143,144], there is no evidence that they can reverse existing disease or the progression of lung function decline; therefore, CFTR modulators may be most effectively utilised as a preventative strategy. This is supported by studies in the G551D-mutated CF ferret model, in which the initiation of IVA treatment in utero resulted in improved pancreatic function and growth and prevented mucus accumulation and bacterial infection in the lungs [145]. Interestingly, when IVA was withdrawn postnatally, multisystem CF disease rapidly developed with similar clinical effects to those also reported in humans after IVA withdrawal [146]. These data form a compelling argument for the early initiation of CFTR modulator treatment to prevent the establishment of CF lung disease entirely [13].

In addition to improving inflammation through moderating host responses, CFTR modulators may have direct anti-inflammatory effects. A recent study showed that IVA/TEZ and IVA/LUM combination therapies not only reduced levels of pro-inflammatory cytokines but also increased levels of the anti-inflammatory IL-10 [119]. Furthermore, IVA has recently been shown to increase NO levels in a small group of CF patients with sino-nasal disease [147]. Therefore, further examination of effects on NO and other anti-inflammatory cytokines or counter-inflammatory molecules is warranted. As proteases and oxidants suppress CFTR function, adjunctive anti-protease and antioxidant therapies may enhance modulator function, particularly in patients with established structural lung disease.

Finally, in this era of disease-transforming therapies, the development of symptom-alleviating therapies to improve airway clearance, chronic infection, and inflammation may seem less important. However, given that not all PWCF will benefit from modulator therapies and as inflammation will proceed particularly in established bronchiectasis, most PWCF are likely to benefit from anti-inflammatory therapies irrespective of CFTR modulator treatment. Therefore, there is an ongoing unmet need for anti-inflammatory therapies in the management of CF lung disease. This has been acknowledged by the CF Foundation, who have established a working group to accelerate the research pipeline for anti-inflammatory therapies [148]. Although several anti-inflammatory therapies are currently in Phase II trials [149,150,151], drug development in this area has been slow despite an abundance of potential inflammatory targets. The lack of well-validated measures of drug efficacy, uncertainty around optimal inflammatory pathways to target, and competition with clinical trials of CFTR-restoring therapies have all been significant challenges for research in the area of inflammation. Furthermore, as the inflammatory state is intrinsically linked to mucostasis and infection, continued development of therapies to improve airway clearance and infection alongside novel anti-inflammatory strategies will be essential in clinical care for the foreseeable future.

## Abbreviations

ASL	Airway surface liquid
BAL	Bronchoalveolar lavage
CF	Cystic fibrosis
CFTR	CF transmembrane conductance regulator
DAMP	Damage-associated molecular patterns
DCs	Dendritic cells
ENaC	Epithelial Na+ channel
ER	Endoplasmic reticulum
FEV_1_	Forced expiratory volume in 1 s
HBE	Human bronchial epithelial cell
IVA	Ivacaftor
LCI	Lung clearance index
LPS	Lipopolysaccharide
LUM	Lumacaftor
MCC	Mucociliary clearance
MDM	Monocyte-derived macrophage
NE	Neutrophil elastase
NO	Nitric oxide
Pa	*Pseudomonas aeruginosa*
PEx	Pulmonary exacerbations
PWCF	People with CF
ROS	Reactive oxygen species
TEZ	Tezacaftor
TRPC	Transient receptor potential channel (TRPC)
